# Structural regulation of PLK1 activity: implications for cell cycle function and drug discovery

**DOI:** 10.1038/s41417-025-00907-7

**Published:** 2025-05-16

**Authors:** Danda Chapagai, Klaus Strebhardt, Michael D. Wyatt, Campbell McInnes

**Affiliations:** 1 Krantz Family Center for Cancer Research, Massachusetts General Hospital, and Harvard Medical School, Boston, MA 02129 USA; 2 Department of Gynecology, University Hospital, Goethe University, Theodor-Stern-Kai 7-9, Frankfurt am Main, 60596 Germany; 3 Drug Discovery and Biomedical Sciences, University of South Carolina, 715 Sumter Street, Columbia, 29208 USA

**Keywords:** Drug discovery, Targeted therapies

## Abstract

Polo Like Kinase 1 (PLK1), a key regulator of mitosis whose overexpression is often associated with poor survival rates in cancer, continues to be widely investigated as an oncology drug target with clinical trials evaluating second and third generation inhibitors. In addition to the conserved N-terminal kinase domain (KD), a unique characteristic of the Polo-Like kinase family is the C-terminal polo-box domain (PBD). The PBD contains a phosphopeptide binding site that recognizes substrates primed by other kinases and furthermore is responsible for subcellular localization of PLK1 to specific sites in the nucleus including centrosomes and kinetochores. Another role of the PBD is its regulatory ability through domain-domain interactions with the KD to maintain an autoinhibited state of PLK1. Insights into post translational modifications and the PBD – KD domain-domain association have been obtained and show that key events in PLK1 regulation include phosphosubstrate binding, T210 phosphorylation and engagement with the Bora protein. These can induce an open and active conformation where the domain-domain inhibitory interactions no longer dominate. Further regulatory events recently described include the interchange between monomeric and dimeric forms, which can also serve to inhibit or activate PLK1 during the cell cycle. Different oligomeric forms of PLK1, existing as homodimers and heterodimers with PLK2, have been identified and likely play context dependent roles. This review provides an overview of recent information describing structural and mechanistic insights into inhibition of PLK1 and the temporal and spatial requirements of its activation and regulation. It also covers recent insights into the conformational regulation of other members of the Polo-Like kinase family. The implications of the conformational regulation of PLK1 with respect to cell cycle function and drug discovery are significant and are therefore discussed in detail.

## Introduction

Polo Like Kinase 1 (PLK1) is only expressed in dividing cells and plays a critical role in several stages of mitosis [1–6]. It is highly expressed in tumors of various origins while its expression is largely absent in surrounding normal tissues [7–11]. PLK1 inhibition can selectively kill cancer cells that are addicted to PLK1 overexpression [7, 12–14]. For example, prostate cancer cells harboring mutations in the tumor suppressor *Pten* [13] or lacking BRCA1 [15] are very sensitive to PLK1 inhibition. PLK1 expression is low throughout G0, G1, and S phase, starts to increase in G2, and rises further in M phase. During interphase, PLK1 is primarily found in the cytoplasm and as levels increase in G2/M, it localizes to the nucleus. The C-terminal polo-box domain (PBD), which is essential for PLK1 localization and function, recognizes a consensus STP motif present on substrates, MAGPMQ-S-pT-P-LNGAKK [16, 17]. In mitotic cells, PLK1 localizes to several sites through the PBD including the centrosome, kinetochore, spindle midzone, centromere and post-mitotic bridge. PLK1 activity is essential throughout mitosis and leads to centrosome maturation, bipolar spindle assembly, M phase entry, sister chromatid cohesion, formation of kinetochore-microtubule attachment, cytokinesis, and mitotic exit [18]. The PBD of PLK1 is recruited through a phosphorylated threonine in the consensus STP, which promotes phosphorylation of substrates by the KD. For example, during prometaphase, PLK1 is involved in regulating chromosome alignment onto the metaphase plate through BubR1 (Budding Uninhibited by Benzimidazole-Related 1 protein) [19]. Priming phosphorylation (by CDK1) of T620 on BubR1 localized on unattached kinetochores creates a PBD docking site resulting in phosphorylation at S676 of BubR1 by the KD [19]. This leads to kinetochore-microtubule interaction, timely mitotic progression, and alignment of chromosomes onto the metaphase plate [19]. Furthermore, Polo-box interacting protein 1 (PBIP1) recruits PLK1 to interphase and mitotic kinetochores and is phosphorylated by PLK1 at T78, leading to self-primed interactions with the PBD of PLK1, but not those of PLK2 or PLK3 [20]. Other key substrates of PLK1 include Cyclin B1 [21, 22], the Translationally Controlled Tumor Protein (TCTP) [23, 24], the phosphatase Cdc25C [25], and Cdh1 [26]. In addition to its canonical mitotic roles, PLK1 has also been shown in recent years to have several functions during interphase. These include regulation of DNA replication [27–29], mTOR signaling [30, 31], apoptosis [32–34], and furthermore in metabolism and the epithelial-to-mesenchymal transition (EMT) of cancer cells.

Since its discovery in *Drosophila melanogaster* [35] and demonstration of its critical roles in mitosis and potential in oncology in humans almost 30 years ago [36, 37] there continues to be intense interest in targeting PLK1. A recent search of Clinicaltrials.gov for “Cancer and PLK1”, indicates 29 studies, 9 of which are listed as recruiting. Despite extensive preclinical investigation suggesting profound efficacy, many ATP competitive PLK1 inhibitors have stalled in clinical trials [38] although recent studies with onvansertib are promising. One reason for the lack of progress may be incomplete mechanistic understanding of the regulation of PLK1 activity and structure, and how this relates to inhibition of the KD, PBD, or both. This review therefore discusses the structural and conformational basis for PLK1 regulation and inhibition and how recent insights will inform next generation drugs targeting this critical cell cycle kinase.

### Structural studies elucidating the domain architecture of PLK1

**Fig. 1:**
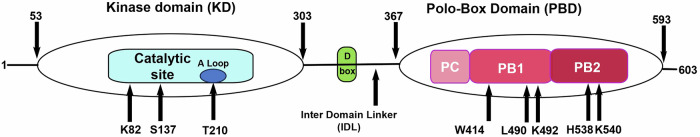
Domain architecture of PLK1 showing KD, IDL region and PBD. The polo-cap is abbreviated as PC and the two polo-boxes are PB1 and PB2. The D-Box and K492 are two sites shown to be involved in PLK1 degradation. Key amino acid residues identified in the kinase domain include the catalytic Lys82 and two sites of regulatory phosphorylation (Ser137 and Thr210). In the PBD, Trp414, Leu490, Lys492, His538 and Lys540 are critical residues for phosphorecognition.

PLK1 consists of two major domains connected by an inter-domain linker (IDL) (Fig. 1) [38–40]. The N-terminal catalytic KD is highly conserved among the PLK family and phosphorylates key substrates in mitosis. The PBD is unique to PLK members and provides the substrate specificity for the PLK family members [6, 41–43]. The PLK1 PBD is comprised of two polo-box motifs (PB1 and PB2, Fig. 1) conserved within related human polo-like kinases and plays critical roles in docking to phosphorylated substrates often primed by other kinases including CDK1 [39]. The kinase activity of PLK1 is regulated through post-translational modifications and through interactions with key partner proteins but also through the PBD, which acts both as an autoinhibitory and as a subcellular localization domain [39].

#### Structural analysis of the PLK1 Polo-Box Domain

The PBD was the first of the two PLK1 domains to be solved crystallographically by two independent groups who generated phosphopeptide complexes [16, 44]. The initial discovery that the PBDs of human, Xenopus, and yeast PLKs all interact with a phosphopeptide recognition motif [17] was then complemented by solution of an experimental 3D structure of the complex of human PLK1 PBD with a consensus phosphothreonine-containing peptide [16]. The structured domain of the C-terminal region of PLK1 was localized from residues 325–603 to 367–603 (Fig. 1) by limited proteolysis and then expression of this construct led to solution of its crystal structure. It revealed that the polo boxes (which despite limited homology with each other are essentially identical when superimposed) form an overall unique fold where they pack together to form a 12-stranded β sandwich flanked by three α-helical segments of PBD that can bind to phosphopeptides [16]. The polo-cap is a structured region at the end of the sequence connecting the KD with the PBD (Fig. 1) and consists of an α-helical segment, loop, 3_10_ helix motif that connects to the first strand of PB1 through a short linker region named L1. The polo-cap tethers PB2 to PB1 and the two polo boxes are connected by another partially conserved 30-residue linker, L2. L1 and L2 are antiparallel and contribute to the hydrophobic core formed by conserved residues from the 4 β-strands of the two polo-boxes.

A phosphopeptide co-crystallized with the PBD binds along a shallow, positively charged groove formed where the two polo box motifs, separated by L2, interact. This peptide, known as the PoloBoxTide [17], is primarily stabilized by ion pairing interactions of the phosphothreonine side chain with His538 and Lys540 but also through an extensive network of bridging structural water molecules. The critical nature of these interactions in defining the molecular recognition of PLK1 with its substrates was confirmed by site-specific mutagenesis (His538Ala and Lys540Met) and the resulting near complete loss of peptide binding to the PBD. The observed exquisite selectivity for serine at the pThr-1 position in substrates is a result of the engagement of this residue with Trp414 and Leu491 through main chain H-bond interactions. Interestingly, in other phospho-peptide complexes, e.g. 14-3-3 proteins [45] and FHA domains [46], the pThr-1 side-chain is solvent exposed and the residue at this position is not conserved [47]. The strict conservation of Trp414, likely due to the serine requirement, confirms why other polo kinase family substrates have a preference for serine and explains the observation that the Trp414Phe mutation eliminates both phosphopeptide binding and centrosomal localization of PLK1 [47]. The observed modest selection for proline at the pThr+1 position and that multiple substitutions in this position are tolerated is confirmed by its lack of stabilizing interactions in the peptide-PBD interface. The proline preference likely results from increased binding affinity through minimized entropic penalty of the cyclin residue. A second independently solved crystal structure [44] largely confirmed the observations of the first study thus providing additional validation of the insights obtained from these experimental structures.

#### Structural determinants of PBD engagement

**Fig. 2:**
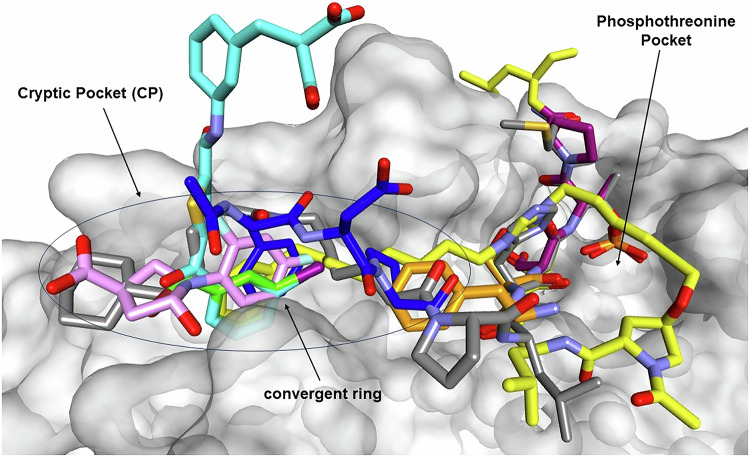
Overlay of PBD-targeted ligands, highlighting the convergence of interactions with the cryptic pocket of PLK1. Five CP engaging ligands (see also Fig. 3A) position an aromatic ring similarly as shown left. Yellow: 4X9R (imidazolium-containing phosphopeptide macrocycle 3B); cyan: 5NEI (polotyrin); green: 5NMM (Alpha-Bromo-3-Iodotoluene); magenta: 5NN2 (Z228588490); grey: 4LKM (PL-74); Blue: 3P7 FDPPLHSpTA, PBIP1 residues 71–79); orange: 5J19 AAFSS[pT]PK, Pon residues 58–65).

The PLK1 PBD recognizes a consensus motif present on its substrates, (i.e., the optimized PoloBoxTide peptide MAGPMQ-S-pT-P-LNGAKK), which is essential for PLK1’s intracellular localization and mitotic functions [17]. The substrate binding site of the PLK1 PBD is inferred from peptide complexes (as no intact substrate/PLK1 complexes have yet been solved experimentally) and contains two subsites identifiable in various inhibitor/PBD complex structures. These are the Ser-pThr binding interface [39] and a hydrophobic cleft that exists as a “cryptic pocket” (CP) i.e., is present only in holo forms (substrate or ligand bound) of the PBD (Fig. 2) [39]. The Ser-pThr subsite consists of residues with positively charged sidechains including His538 and Lys540 to engage the negatively charged pThr with high complementarity and affinity. As stated, mutation of these critical residues is known to disrupt PLK1 binding to phosphorylated substrate proteins and thus abrogate many PLK1 functions [16]. To date almost 50 peptide and peptidomimetic crystal structures have been obtained and even where the rest of the PBD ligand diverges structurally, the interactions of the serine and phosphothreonine in each of these structures converge and have almost identical interactions with Trp414, His538 and Lys540 [39]. A key study identified minimal phosphopeptides based on PBIP1 that retained potent and highly specific binding for the PLK1 PBD binding (a 5mer) with even a tetrapeptide retaining activity [48]. Baxter et al. carried out further SAR on the PBIP1 and cdc25C sequences and identified chimeric sequences with exquisitely potent affinity for the PLK1 PBD [49].

The hydrophobic cleft (labeled cryptic pocket, Fig. 2) was first discovered in PBD structures where part of the substrate binding groove was shown to engage the lipophilic component of a peptidomimetic containing alkylated histidine sidechains [50–53]. As this subsite was not apparent in initial peptide structures, it opens up when bound to appropriately substituted PBD inhibitors and thus can be considered a cryptic pocket (CP). Due to the presence of four tyrosine residues (conserved across the eukaryotic members of the polo-like kinase family) and as a result of its adaptability to substrate and ligand binding, it is also known as the “Tyr pocket” [41, 54]. The structures revealed that these tyrosine residues (417, 421, 481 and 485) form the sides of the CP and Val415, Leu478 and Phe482 make up its base. The major rearrangement that occurs upon CP ligand binding is the rotation of the sidechains of Tyr417 and Tyr421 which no longer block access to this pocket.

**Fig. 3:**
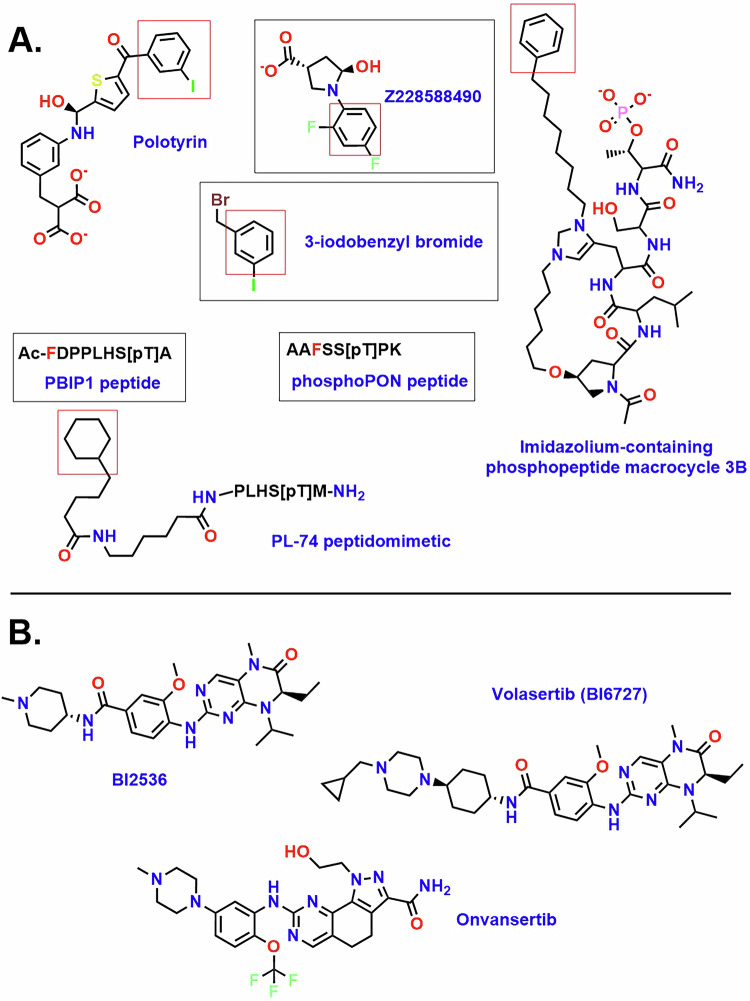
Chemical Structures of PLK1 Inhibitors. **A** PBD-targeted ligands, that engage the cryptic pocket of PLK1. Diverse ligands including peptides, peptidomimetics and small molecules that bind to the PBD are shown. The convergent ring engaging the CP (Fig. 2) is highlighted in the red boxes and letters for peptides. **B** ATP competitive inhibitors of the PLK1 kinase domain.

The Tyr pocket revealed in PBD peptidomimetic structures was subsequently shown to play a role in a subset of PLK1 protein-protein interactions and evidence was provided that the cryptic site directs PLK1 to specific substrates [41]. Polo-box interacting protein 1 (PBIP1) is essential for recruiting PLK1 to interphase and mitotic kinetochores. PLK1 phosphorylates PBIP1 at Thr78 and then binds to the resulting S77-pT78 motif via the PBD. The pThr78 PBIP1-dependent PLK1 localization to kinetochores is required for chromosome congression and spindle assembly checkpoint [20]. pThr78-containing peptides interact with the PBD of PLK1 but not with those of PLK2 and PLK3, suggesting an additional level of regulation through the specificity of the interaction [20, 49]. Crystal structures of PLK1 PBD complexes with FDPPLHSpTA (PBIP residues 71–79, PDB: 3P37) or DPPLHSpTA (PBIP residues 72–79, PDB: 3P36) revealed that Phe71 of PBIP1 has context dependent interactions with Tyr417 and Tyr421 of the Tyr pocket [54]. In the DPPLHSpTA-PBD structure, the hydrophobic pocket adopts a closed conformation and does not interact with the shorter peptide, while in that of the FDPPLHSpTA-PBD complex, Phe71 inserts into the hydrophobic slot and induces the CP with tyrosines 417 and 421 opening up (see blue colored peptide in Fig. 2 and also in Fig. 3A) [54]. This work also explored the role of Phe71 using thermal shift analysis and isothermal titration calorimetry and revealed that it plays a significant role in the affinity of PBIP1 for PLK1. Further studies from a different group made use of *PLK1*-depleted cells overexpressing either GFP-PLK1_AAD_ (mutation of Tyr421Ala, Leu478Ala and Tyr481Asp) or GFP-PLK1_AM_ (mutation of the pThr interacting residues i.e. His538Ala, and Lys540Met) [41]. Both mutant proteins showed impaired PLK1 localization to kinetochores but not to the centrosomes. These results suggest that the CP is functionally important for recognition of a subset of PLK1 substrates, specifically with those required for PLK1 localization to kinetochores including BubR1, PBIP1, CLASP2 and CLIP-170 [19, 20, 55, 56]. HeLa cells overexpressing GFP-PLK1_WT_ were transfected with either PBIP1-V5 (V5 was C-terminally tagged to protein), PBIP1-Phe71Ala-V5, PBIP1-Thr78Ala-V5 and pull-down studies carried out. Both mutants weakly bind to GFP-PLK1_WT_ compared to wildtype PBIP1-V5 suggesting that Phe71 and Thr78 are essential for the PBIP1-PLK1 interaction during mitosis. Further experiments revealed that GFP-PLK1_AM_ fails to bind NEDD1, while the GFP-PLK1_AAD_ retains binding to this key PLK1 substrate [41]. These experiments provide strong evidence that the Tyr cryptic hydrophobic pocket can act in concert with the Ser/pThr binding cavity to specifically recognize a subset of PLK1 substrates [41]. Depletion of endogenous PLK1 causes monopolar spindle arrest with misaligned chromosomes. Additional studies explored the consequences of inducing expression of GFP-PLK1_WT_ (restores both bipolar spindle formation and chromosome congressional defects), GFP-PLK1_AAD_, GFP-PLK1_AM_ (both restored bipolar spindle formation but do not counteract the chromosome congressional defects) in the context of depleted PLK1. These results suggest that both the Ser/pThr and Tyr pocket subsites of the PBD are essential for the chromosome congression but not for the bipolar spindle formation [41]. The role of the CP in selectivity of PBD ligands for PLKs 1–5 has not been extensively investigated and further studies into this are warranted.

In addition to the PBD complex structures for CP binding peptides and alkylated peptidomimetics, several small molecules have been identified that exclusively engage the CP and do not interact with the pThr binding site [41]. These compounds insert effectively into the CP subsites that components of the peptidomimetics bind to. Polotyrin (aka JES107, Figs. 2 and 3A) contains a 3-Iodobenzoyl)thiophene structure where the iodophenyl overlays closely with the phenyl ring from a imidazolium-containing phosphopeptide macrocycle and interacts with tyrosines 417, 421 and 481. Z228588490 (Figs. 2 and 3A) is a small molecule containing a bis(fluoranyl)phenyl]-5-oxidanyl-pyrrolidine and whose crystal structure reveals that the difluoro phenyl substituent inserts similarly to that seen with the peptidomimetic phenyl ring [41]. The complex of the simple ligand Alpha-Bromo-3-Iodotoluene (Figs. 2 and 3A**)** with the CP also reveals a similar binding mode of the aromatic ring [41]. This structural convergence suggests that this aromatic ring is a key determinant of PBD engagement.

Due to its unique engagement of the CP, polotyrin was further investigated in cellular experiments and these were shown to be consistent with the mitotic roles of PLK1 [41]. However, it should also be noted that the in vitro IC_50_ value for polotyrin binding to the PLK1 PBD was >100 μM, and cellular experiments with polotyrin showed minimal induction of mitotic arrest at 1 mM polotyrin [41], indicating suboptimal binding to the CP and the likelihood that non-specific binding plays a role in this compound’s activity. Furthermore, its ability to influence subcellular localization of PLK1 or the kinase activity was not explored. Overall, the CP has been demonstrated to be a critical component in the recognition of substrates by PLK1 and as novel mode of inhibiting activity through the PBD.

#### Structural analysis of the PLK1 kinase domain

**Fig. 4:**
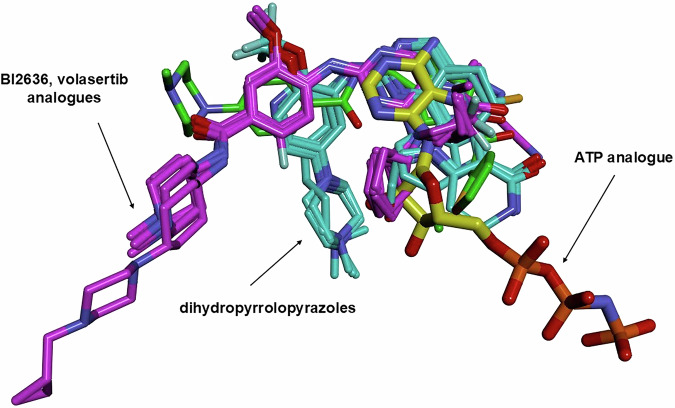
Overlay of ATP competitive inhibitors of PLK1 obtained to date and highlighting the distinct binding modes observed. The binding mode of an ATP analogue with carbon atoms highlighted in yellow is shown for reference. Magenta represents BI2536, volasertib and similar ligands; cyan represents onvansertib and similar ligands; green (2OWB) is a 1,5-dihydropyrrolo[3,4-c]pyrazole derivative.

The first crystal structure of the KD was solved with the impetus for its study primarily coming from the interest in developing ATP-competitive inhibitors of PLK1 as potential anticancer agents [57]. The crystal structures of the Thr210Val mutant of the KD of human PLK1 complexed with a nonhydrolyzable ATP analogue and the pyrrolo-pyrazole inhibitor PHA680626 (Fig. 4) were solved to high resolution. The catalytic domain was shown to adopt a typical kinase domain fold in an active conformation and where the ATP binding site formed between the N-terminal lobe (residues 37–131), composed predominantly of an antiparallel β-sheet, and the primarily α-helical C-terminal lobe (residues 138–330). As with other kinases, the two lobes were shown to be connected by a hinge region and which undergoes critical interactions with both ATP and inhibitory compounds. Continuous electron density for residues 37–330 was observed (14–36 and 331–345 had none). Residues 43–46 made up the β strand preceding the first strand of the kinase domain core as defined for other kinases. Residues 306–330 were shown to be structured and comprised a section of the linker connecting the KD and PBD. This structure also provided the first picture of the PLK1 ATP binding pocket and demonstrated that the interactions of ATP (specifically AMP-PNP, a non-hydrolysable analogue) were similar to nucleotide binding in other kinases. Specifically, the adenine ring N1 hydrogen bonds to Glu131 and Cys133 of the hinge region. The pyrrolopyrazole analog PHA680626 structure showed a similar orientation of its core structure to the adenine, with it making equivalent hinge hydrogen bonds and being sandwiched between Cys67 and Phe183 such that its methylpiperazine group is solvent exposed. Other interactions of PHA680626 include water-mediated hydrogen bonds with the catalytic Lys82 and His105 and hydrophobic contacts with the gatekeeper residue Leu130 in the back of the pocket. These structures revealed unique features of the PLK1 active site including the unusual Cys67 and several positively charged residues adjacent to the hinge region, suggesting how they could be exploited in the design of selective inhibitors [58]. Subsequent to this initial structure, numerous other complexes of compounds specific for the PLK family have been solved with the isolated KD [58–67]. At date of publication of this review, 15 KD inhibitor complexes have been deposited in the PDB and reveal the structural basis for potent and selective inhibition of catalytic activity [38]. Of these structures two major binding modes were observed (Fig. 4) and cluster around the positioning of BI2536 and onvansertib (Fig. 3B), two clinically evaluated PLK1 inhibitors. Interestingly, superimposing all these structures confirms that very little variation in the backbone conformation occurs and that there is no major structural induction or conformational change that occurs upon ligand binding.

Further conclusions of the above study from Kothe et al. shedding light onto the activation of PLK1 indicated that while mutations of Thr210 in PLK1 have been shown to change PLK1 kinase activity and affect PLK1 function in vivo, regulation of PLK1 in the cellular context also relies on interactions of the PBD with the KD, with regulatory partners, as well as oscillation of its levels throughout the cell cycle. They surmised due to the observation that the Thr210Val PLK1 crystallized in the active conformation that the activation loop conformation does not need to be as tightly controlled in PLK1 as in other kinases. Further structural observations regarding the PLK1 KD were that two of the residues that contribute to the RD pocket (the catalytic Asp is preceded by an invariant Arg) are not conserved in PLK1 (Met100 and Thr199, corresponding to Asn90 and Lys189 in Protein Kinase A). In RD kinases, the arginine creates a basic patch that helps neutralize the charge of the phosphorylated activation loop and stabilizes the active conformation [68]. In the unphosphorylated PLK1 context, reduced electrostatic repulsion within the RD pocket may allow for the active state population to be increased relative to other kinases.

### PLK1 PBD dimerization regulates PLK1 kinase activity

Phospho-peptide binding to the PLK1 PBD is known to partially activate PLK1 most likely by relieving an autoinhibited conformation, for which a significant body of evidence exists [69]. Another mechanism through which PLK1 activity has been shown to be regulated is through dimerization events, the details of which have been revealed in recent studies. Zhu et al., demonstrated that dimerization of the PLK1 PBD occurs, using biochemical and in vitro assays but mainly through a crystal structure of the PBD in complex with the phosphoPON peptide [70]. CDK1 phosphorylates the Drosophila protein Pon (adaptor Partner of Drosophila Numb) at Thr63 thus creating a PLK1 PBD docking site. In this study, comparison of the PBD dimer with that of the autoinhibited PLK1 structure suggests that phospho-Pon (pPon) peptide binding relieves autoinhibition occurring through the closed conformation and this occurs through the formation of stable PLK1 dimers. Binding between pPon and the PBD is mediated by polar and hydrophobic interactions. In the crystal structure of the PLK1 PBD-pPon complex, Ser62 of pPon forms a hydrogen bond with the backbone of Trp414 and the phosphate group of pThr63 of pPon has the typical charge-charge and hydrogen bond with Lys540 and His538 respectively. In addition to these polar interactions, hydrophobic contacts promote the PBD-pPon binding and furthermore stabilize the dimeric PBD interestingly through the Tyr pocket described in detail above (Figs. 2, 3A). The sidechain of Phe60 of the pPon peptide inserts into the hydrophobic cleft (CP) formed by Val415, Tyr417, Phe482 and Tyr485 in an analogous fashion to Phe71 of PBIP1 described above. The dimerization interface is mediated by extensive hydrophobic interactions including the side chain of Leu505 from L2 of one of the PBD molecules in the dimer. This residue binds to the cryptic pocket of the other PBD monomer which also includes the Phe60 pPon peptide side chain. The interaction between PBD-bound Phe60-pPon and Leu505 from one PBD is therefore proposed to stabilize dimeric PLK1. As expected from the observed critical roles of both residues in the dimerization interface, the point mutant Leu505Glu abrogated formation of the dimer complex as shown by static light scattering studies (SLS). PLK1 PBD elutes as a major peak corresponding to a monomer although the dimer exists as a minor peak. The PBD in the presence of pPon elutes as a heterogeneous mixture of monomers and dimers but the majority of the proteins exist as dimeric forms. PBD Leu505Glu elutes as a 30 kDa peak and is thus consistent with a monomeric PBD. Intriguingly, changing a Phe to Ala of pPon (pPonPhe60Ala) inhibits PLK1 kinase activity, which is opposite to the effect mediated by pPon. This provides further evidence for the role of peptide mediated dimerization in mediating PLK1 catalytic activity.

With regards to further roles of PLK1 dimerization, a recent study indicated that it plays a role in mitotic recruitment to kinetochores [5]. Phospho CENP-U (the PLK1 receptor in the kinetochore core) was shown to promote PLK1 docking through dimerization. In this study the requirements for recruitment of PLK1 to Thr78 and Thr98 of CENP-U were investigated and how these sites were potentially involved in recruiting multiple PLK1 molecules. Fluorescently labeled MBP-PLK1(Thr210Ala) and CENP-OPQUR were incubated in the presence of CDK1 and PLK1 and the resulting complex examined by size exclusion chromatography and sedimentation velocity analytical ultracentrifugation. The MBP-PLK1Thr210Ala:CENP-OPQUR complex had a sedimentation coefficient and an apparent molecular mass consistent with the formation of a complex of two PLK1 molecules and CENP-OPQUR. These results indicate that two molecules of PLK1 bind phosphorylated CENP-U. After recruitment of the first PLK1 molecule, which binds to pThr98 created by CDK1 priming, stable binding of dimeric PLK1 results after local phosphorylation of the neighboring site Thr78 by the first PLK1 to bind. The second PLK1 molecule is then recruited to pThr98 through its PBD thus creating dimeric PLK1 bound to CENP-U. Dimerization of PLK1 through the PBDs explains why both CDK1 and PLK1 are necessary for complex formation with CENP-U in that CDK1 provides the initial priming phosphorylation and a PLK1 molecule provides a priming phosphorylation to recruit the second PLK1 in the dimer. This study also proposed a model of PLK1 dimerization relevant to formation of the BUB1:BUBR1 heterodimeric complex required for the recruitment of PLK1 to kinetochores. Phosphorylation of BUB1 and BUBR1 by CDK1 kinase, on Thr609 and Thr620 respectively, occurs prior to PLK1 recruitment, and this leads to dimerization of PLK1. Sequential CDK1-PLK1 phosphorylation has been observed with various PLK1 substrates, including NEK9 [71], NEDD1 [72], Caspase8 [33], and CDC25 [73]. Furthermore, MKLP2, another self-primed substrate of PLK1 (in anaphase), has tandem Ser-Thr-X motifs of similar spacing to CENP-U [74]. This study suggests that there is a firm requirement for adjacent motifs to facilitate dimerization, however the order of phosphorylation is not rigid. This study also indicated that several other known or potential PLK1- binding partners, including Meikin [75] and BuGZ [76], have constellations of CDK1 and PLK1 sites that closely resemble those of BUB1 and CENP-U.

**Fig. 5:**
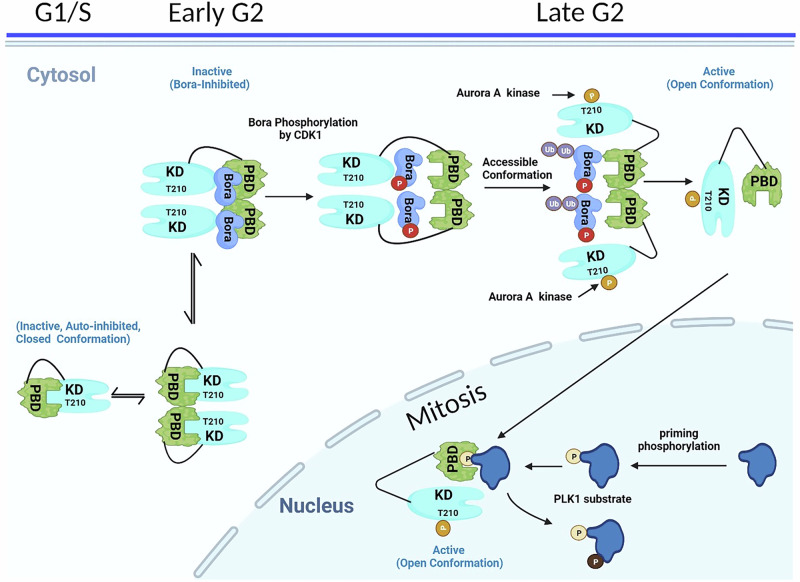
Monomer-dimer regulation of PLK1 activity [77]. PLK1 assumes an inactive closed and autoinhibited structure in G1 and S phases of the cell cycle. To fully sequester in an inactive state, Bora binding stabilizes dimeric PLK1 which is maintained until late G2 when Bora degradation and Aur-A phosphorylation results in a monomeric and open conformation of PLK1 which is fully activated both in terms of its catalytic activity and ability to bind to and recruit substrates in specific subcellular locations. Monomeric PLK1 has an exposed nuclear localization signal which then facilities its translocation into the nucleus. Figure was created using Biorender.

A further recent study looked exclusively at the role of dimerization of PLK1 in its activation and regulation in the cell cycle [77]. This in-depth analysis was undertaken to investigate the molecular mechanism by which Thr210 in cytoplasmic PLK1 exists in its unphosphorylated state during early G2 even when its activators, Aurora Kinase A (Aur-A) and Bora are present [78–80]. Using a variety of techniques including co-immunoprecipitation, fluorescence resonance energy transfer, Nanobit, crosslinking and size exclusion chromatography studies, Raab et al. determined that Bora transiently promotes dimerization of cytoplasmic PLK1 during the entire G2 phase of cell cycle (Fig. 5). Contrary to the Zhu et al. study which primarily involved biochemical studies [70], this group determined that monomeric PLK1 differs from dimeric in terms of catalytic activity and that dimerized PLK1 sequesters kinase activity until required. Phosphorylation of PLK1-Thr210 by Aur-A in late G2 results in loss of PLK1 dimerization and therefore produces the active monomeric PLK1 for its roles in G2 and mitosis (Fig. 5). As pThr210 disrupts homodimerization, this indicates that the kinase domain itself is at least partly involved in the dimerization. Although the Zhu et al. study confirmed PBD-mediated PLK1 homodimerization, Raab et al., also showed evidence for PBD mediated dimerization and that it is likely that both domains are involved. The role of Thr210 was further corroborated by expression of the phosphomimetic mutant PLK1-Thr210Glu, which disrupted dimer formation and led to monomeric PLK1 [77]. Time kinetic experiments demonstrated a partitioning of monomeric and dimeric PLK1 between cytoplasm and the nucleus in that monomeric protein is translocated into the nucleus to carry out its mitotic activities. Dimeric PLK1 in G2 reduced the association of PLK1 with importin, decreased nuclear levels and this in turn resulted in mitotic consequences including aberrant cytoplasmic accumulation of PLK1. Blocking the G2 PLK1 dimer-to-monomer switch hinders its nuclear transport and thus blocks PLK1 access to mitotic subcellular structures. Studies suggest that PLK1 is maintained in a closed conformation within the PLK1-Aur-A-Bora complex [81]. Furthermore, the N-terminal domain of Bora in complex with PLK1 is subject to multiple phosphorylation events that modify PLK1 conformationally [81]. At the end of G2, these conformational transitions might lead to a dimeric form but now with an accessible T-loop, thus enabling PLK1 activation and hence leading to degradation of Bora (Fig. 5). Collectively, these events promote the release of the dimers and switch PLK1 to the monomer active form, facilitating importin-dependent nuclear entry during early mitosis. In their model, PLK1 dimerization is a mechanistic feature enabling the fine-tuning of its activation at the end of G2 and into mitosis.

Heterodimerization between PLK family members may also play important roles in cell cycle regulation and provide therapeutic opportunities. A recent study demonstrated that PLK2 directly interacts with PLK1 at prometaphase primarily through the kinase domain and not through the polo-box domain of PLK2, suggesting that PLK2 activity is at least partially dependent on PLK1 [82]. PLK2 was shown to be a tumor suppressor in breast cancer (basal-like and triple-negative breast cancer subtypes) and loss of PLK1 rescued phenotypes after *PLK2* knockdown in vitro and in vivo. Interestingly, the use of the PLK1 inhibitor volasertib alone or in combination with carboplatin resulted in greater efficacy in preclinical models of TNBC (with low levels of PLK2) whereas re-expression of PLK2 in an inducible PLK2-null mouse model reduced response to volasertib. The authors propose that low PLK2 expression might be a predictive marker for PLK1-targeted therapeutics such as volasertib, which has good selectivity for PLK1 over PLK2. It is interesting to speculate on these observations as they impact PLK1 dimerization and function. One interpretation could be that a PLK1/PLK2 heterodimer promotes resistance because the heterodimer is not inhibited to the same extent that a PLK1 monomer or homodimer is, and thus promotes the mitotic functions of PLK1. An alternative interpretation might also be that exogenously overexpressed PLK2 can directly substitute for at least some functionality of PLK1 required for mitotic progression. Either interpretation requires more extensive future study.

### Allosteric mechanisms of PLK regulation

As mentioned above, while a plethora of structural information on the independent kinase and polo-box domains exist, obtaining a crystal structure of full length (FL) PLK1 has been elusive to date. It is likely that the multiple conformational states and flexible regions preclude crystallization. A creative solution was to investigate the complex of the isolated domains and it was found that a crystal structure (PDB ID: 4J7B) [69] could be obtained using polo-like kinase KD and PBD from zebrafish (*Danio rerio*). This complex was generated in the presence of a Map205 polypeptide (PBD-binding motif of microtubule-associated protein 205), a protein unique to *D. melanogaster*. The ternary complex provided considerable insights into how PLK1 activity is regulated and revealed that the KD is inhibited by the PBD with a large contact interface observed between the two domains. The overall conformation of the KD is similar in both the isolated domain and in this autoinhibited complex. However, in the latter, rigidification of the hinge region and sequestration of the activation loop prevents conformational adaptations required for full kinase activity. Furthermore, an extended region of the PBD makes close contact near the ATP binding site and occludes the catalytic cleft. Based on this KD–PBD complex and biochemical data from this study, mechanistic conclusions were made. Firstly, activation of PLK1 likely occurs by releasing this autoinhibited confirmation. PLK mutants were introduced into the KD/PBD interface to destabilize the interactions between the two domains. P394K (P403 human) and V399R (I408 human) mutations were shown to significantly increase kinase activity [69]. The conclusion was also made that the IDL inhibits the KD and sequesters T196 (T210 human) from phosphorylation. To further explore how this might occur, the IDL was modeled into the crystal structure (absent due to the structure being two independent domains) and determined that it is of sufficient length to connect the C-terminus of the KD and the N-terminus of the PBD, which perturbs the autoinhibited structure. It was thus found to be similar to other autoinhibited kinases such as the Src-family [83, 84] and focal adhesion kinases [85]. Their modeled structure showed that the IDL directly contacts the activation loop and therefore interferes with Aur-A phosphorylation of T196. Xu et al. however, did surmise that their proposed IDL mechanism might differ with respect to the way human PLK1 is regulated in different organisms as the IDL from various species have diverse sequences and lengths [69]. They also cautioned in extrapolating conclusions from the somewhat artificial KD–PBD–Map205PBM heterotrimeric complex to different organisms on the basis of this crystal structure. At the same time however, as residues of the PBD interacting with Map205PBM and at the interface of the KD and PBD are highly conserved among humans, zebrafish and *Drosophila*, then it is highly likely that their conclusions are more broadly relevant. Indeed, mutation of analogous residues in human PLK1 resulted in activation although to somewhat different degree than that observed with the Danio protein used for the crystal structure [69]. This may be attributed to more diverse IDLs despite the high conservation of the interfacial residues between the KD and PBD of PLK1s. Xu et al. proposed as a result of this work and conclusions drawn from other mechanistic studies of PLK1 phosphorylation, binding partners, and substrates, that there are a number of conformational transitions that can occur between the autoinhibited PLK1 and its fully activated state. Partially active PLK1 states occur separately through phosphorylation of S137 leading to PBD relief, T210 phosphorylation leading to IDL relief and further phosphopeptide binding to the PBD leading to PBD relief [69]. This model almost certainly needs refining because of recent insights into the involvement of Bora and homodimerization of PLK1 and the fact that some of the intermediate activated states may be related to the dimer-monomer transition which occurs in late G2 and M phases [77].

A recent study investigated FL PLK1 using multiple biophysical techniques and data integration for structural modeling of PLK1 [40]. This was carried out to generate additional insights into the domain-domain interactions and regulatory conformational changes occurring in PLK1 realizing the limitations of the artificial Danio KD/PDB complex that lacked the IDL and included the map205 protein only relevant in *Drosophila*. Integration of SAXS (outlines the overall domain arrangements) and hydroxyl radical protein foot printing (HRPF, used to probe the solvent accessibility of residues on the surfaces of individual domains) experiments were integrated in conjunction with conformation sampling and molecular modeling to derive a potentially more relevant model of the KD–PBD complex. From this study by Ruan et al., a compact yet elongated domain-arrangement was observed and revealed a network of interacting residues at the KD–PBD interface where the PBD interacts with the kinase domain C-lobe. Specifically, evidence was found for the interaction of K143 of the KD and L491 close to the phosphopeptide binding site. These residues were shown to be highly solvent-exposed in their individual domains but are sequestered from solvent after domain-domain assembly. These observations were further supported by mutations of these two residues in that activity of PLK1 was increased after individual interchange and significantly more so after both mutants were combined. From the complex generated by molecular dynamics, other residues in this potential interface were mutated, such as E488K and K492A and also demonstrated to increase PLK1 kinase activity. While evidence for this domain-domain interface is strong, the results contrast with the interface observed in the Danio complex which was supported by a different set of mutational data and by the recent analysis of the Alphafold FL PLK1 structure (following discussion in this section). Ruan et al. did not however consider the possible influence of PLK1 dimerization in their data interpretation. As discussed above, various PLK1 dimer forms have been observed and it is possible that their novel observations have identified residues involved in intermolecular interactions, i.e., from the KD of one monomer to the PBD of another monomer and not intramolecular as proposed. The authors do state that the FL human PLK1 adopts dynamic structures with a variety of domain-domain interfaces in solution and therefore it is possible that the conformation identified is relevant under specific circumstances.

While not providing direct structural evidence, other mechanistic studies have provided insights into the conformational transitions of PLK1 from its autoinhibited (closed) to active (open) states and thus of allosteric regulation. One study examined the interactions of the kinase inhibitors BI2536, Volasertib (BI6727) and RO3280 with their primary target PLK1 using fluorescence spectroscopy and molecular dynamics calculations as tools to probe their chemical biology [86]. Observation of high Stern-Volmer constants from fluorescence experiments indicated that stable protein-ligand complexes were formed with each of these inhibitors. The binding constant between BI2536 and PLK1 was found to increase approximately 100-fold in presence of a Cdc25C-p phosphopeptide that is known to interact with the PBD. These results suggest that the affinity increase is consistent with the studies described above indicating that BI2536 binding leads to release of the autoinhibited structure and leads to a catalytically inactive but open form. That said, this study lacked a certain amount of rigor in that only BI2536 was compared in the presence and absence of the phosphopeptide and furthermore that it was shown to have a relative affinity only 6-fold higher compared to ATP. BI2536 is known to have a low nanomolar IC_50_ for kinase inhibition of PLK1 and this observation is hard to reconcile, because studies have previously shown this compound has at least 1000 times higher affinity for PLK1 than ATP [87]. This work needs clarifying by systematically comparing the K_a_ values for matched data sets in the presence and absence of the cdc25C phosphopeptide and by measuring affinities using an alternate by complementary biophysical techniques.

Using an Alpha Screen assay, Raab et al. discovered small molecules that enhance or inhibit the interaction between the PLK1 PBD and a phosphorylated peptide (PoloBoxtide, Biotin-MAGPMQSp[T]PLNGAK) [88]. One small molecule, FM0024, enhanced the interaction between FL PLK1 and PoloBoxtide while SCR01010 and AW00551 diminished the interaction between PoloBoxtide and FL PLK1. As none of these compounds directly inhibit the interaction between the isolated PBD and PoloBoxtide, it is likely that they allosterically modulate the activity of PLK1. In in vitro kinase assays, these three compounds all inhibit the kinase activity of both FL PLK1 and the isolated KD but only AW00551 did so equally in both constructs. Further studies were carried out with known validated PLK1 inhibitors. An in vitro thermal stability assay showed that BI6727, which strongly stabilizes the KD against heat denaturation, conversely caused a distinct biphasic pattern in the melting curve with an initial destabilization. These experiments provide evidence that the binding of BI6727 destabilized FL PLK1 at lower temperatures but leads to its stabilization at higher temperatures. One possible explanation for these observations is that BI6727 binding to the KD relieves the compact autoinhibited form, generating the open conformation which is less thermally stable. In agreement with this result, BI6727 enhanced the interaction between the PBD of PLK1 and PoloBoxtide in the AlphaScreen assay, which also suggests that its binding to the KD is promoting an open conformation of PLK1, exposing the PBD phosphopeptide binding site. FM0024 mildly stabilized the KD of PLK1 but not FL PLK1 and also induced a distinct PLK1 denaturation pattern in which the first part of curve indicates stabilization, and the second indicates destabilization. In agreement with the findings from biochemical assays, FM0024 augments the formation of the PLK1-Bora complex, which primarily occurs through the PBD. Cells treated with FM0024, which enhanced the interaction between the PBD of PLK1 and PoloBoxtide, undergo enhanced phosphorylation of PLK1-specific substrates including TCTP, Myt1, Cdc25c in HeLa cells. Another identified compound AW00551 was shown to stabilize FL PLK1 and the isolated KD but decreased the interaction between the PBD of PLK1 and the PoloBoxtide in the AlphaScreen assay and furthermore reduced the phosphorylation of Myt1 in HeLa cells. In summary, Raab et al. described how small molecules with different modes of action and varying binding sites on PLK1 can produce varying outcomes in cells and suggest that allosteric binders provide insights into mechanisms of inhibition and conformational states of PLK1.

Chapagai et al. performed complementary studies using fluorescence polarization and cellular thermal shift (CETSA) assay. The results demonstrated that BI2536 substantially increased the affinity of a PoloBoxtide-derived tracer and furthermore that non-peptidic compounds also bound tighter. Moreover, it was shown that treatment with BI2536 or BI6727 (volasertib) thermally *destabilized* PLK1 in the cellular context as measured by CETSA [89]. These studies are consistent with ATP competitors inducing an open but catalytically inhibited form of PLK1. Such a conformation, while inactive, may therefore promote non-catalytic activities and therefore have significant implications for drug discovery and development based on inhibiting the ATP binding site of PLK1.

### Insights into regulation of Polo-kinase family members using AlphaFold

**Fig. 6:**
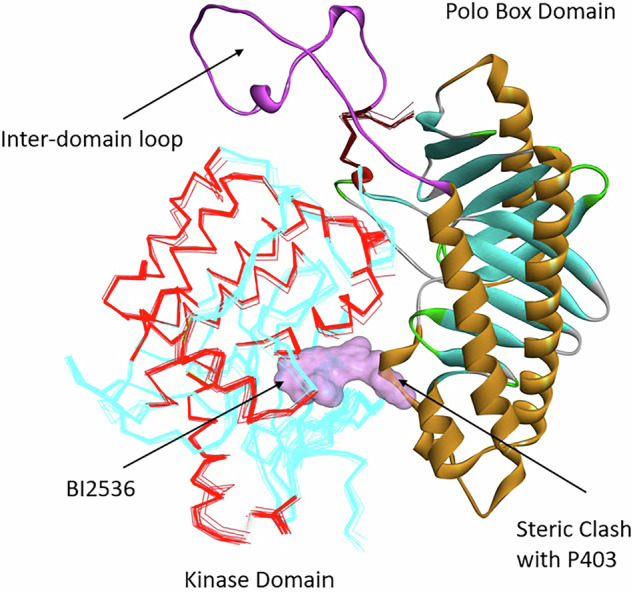
Autoinhibited structure of PLK1 generated using AlphaFold [93]. The structure generated was found to be in the autoinhibited, closed conformation state, and shows the IDL (purple) bridging the KD and PBD, a key detail absent in the Danio structure [69]. The kinase domain represents the overlay of the co-crystal structure of the isolated KD structure bound to BI2536. The two polo boxes of the PBD are shown in ribbon form. When the KD inhibitor BI2536 (lavender surface) was modeled into the active site, a severe steric clash occurs with Pro403 of the PB1 of the PBD. Experimental data strongly suggests that this results in an open but catalytically inactive conformation of PLK1.

To provide further insights into human FL PLK1 activation, the structure of FL PLK1 was analyzed using AlphaFold (AF) [89, 90], an AI algorithm shown to be highly successful and accurate in generally predicting protein structure [91–94]. Analysis of the human FL PLK1 structure generated by AF in comparison with available crystal structures of individual KD and PBD domains revealed some remarkable observations. The AF structure shared high similarity with the Danio Rerio PLK1 structure (isolated KD and PBD co-crystallized, discussed earlier [69]) but provided new insights absent in the artificial complex. The AF predicted structure was found to be in the autoinhibited state (Fig. 6) and when overlayed with the Danio protein complex, shown to be almost identical. The observed similarities therefore provide additional validation for a conserved regulatory mechanism in diverse polo kinase family enzymes. As AF generally seeks to find low energy states of proteins, this result also suggests that the autoinhibited conformation is the most populated structure in the absence of post-translational modifications and ligand binding. The predicted human FL PLK1 structure confirmed the extensive interface between the KD and PBD stabilized largely by buried hydrophobic residues. The PBD residues contributing to the closed conformation include the 403–407 loop of PB1 that is in close proximity to the ATP binding site of the KD. I408 and F409 make extensive contacts to the C-terminal region of the KD including residues 316–320. L505 has extensive contacts to the KD and contributes the most to stabilizing the closed conformation. Additional residues from PB2 that contribute to stabilization of the autoinhibited state include L546 and M547 which contact residues 140–146 of the KD and are close in proximity to the P403 loop adjacent to the ATP binding site.

Absent in the Danio structure but known to participate in regulation of PLK1 activity is the 50-residue sequence (residues 315–365) connecting the kinase domain with the PBD polo-cap. This sequence comprises the Interdomain Connecting Loop (IDL) and acts as a hinge allowing opening of the two domains. The AF structure suggests that the IDL is partially structured and contains a one helical turn. It also confirms that the α-helix of the PC can be extended almost two more turns (365–387, equivalent residues 365–371 missing in 47B). Further, it reveals the missing segment from residues 489 to 498 in the Danio complex where L490 interacts with M486 and Y485, residues that are part of the cryptic pocket, and thus likely contribute to PBD substrate recognition.

Analysis and overlay of an isolated KD structure bound to BI2536 with the FL PLK1 AlphaFold structure revealed that the autoinhibited, closed conformational state is not compatible with BI2536 binding to the ATP-binding pocket, as severe steric clashes result when this ligand binds to FL PLK1 (Fig. 6) [89]. In particular, the piperazine ring of BI2536 overlaps with P403 of the PBD, suggesting that BI2536 binding significantly perturbs the FL PLK1 structure and precludes the autoinhibited conformation of PLK1. This is therefore consistent with increased binding to the PBD, supporting the observations that this and its related analog BI6727 induce an open conformation of PLK1 in which the PBD is exposed and allows tighter binding of its ligands [88, 89]. Analysis of peptide-bound, isolated PBD domain structures overlaid with the FL PLK1 AlphaFold structure provided insights not found in the Danio autoinhibited PLK1 and reveals that steric clashes also occur in the PBD [89]. These are consistent with the reduced affinity of peptides for FL PLK1 compared to PBD fragments and furthermore for the increased affinity of the peptide tracer and a non-peptidic abbapolin inhibitor. For the most part, the PBD structures are very similar except for the linker 2 region (L2 – human residues 490–502), which is significantly different in the two structures. As L2 exits from PB1 (C-terminal to PB1), the two L2 regions start to diverge. It is exactly this region that contacts residues of PBD-interacting proteins that are C-terminal to the pThr. The sidechain of Leu6 of the hexapeptide in 1Q4K interacts with the end of the PB1 α-helix and with the sidechain of Leu491. The overlay of the PBDs shows that in the AF structure, there is a significant clash with the peptide in the inhibited state and therefore a conformational adaptation would be required to accommodate peptide binding. This steric clash is consistent with the observation of reduced peptide affinity in the absence of BI2536 since FL PLK1 exists in the inhibited state where the L2 loop interferes with peptide binding. It is likely that the isolated PBD bound to the peptide more closely represents the conformation of L2 that exists in the open conformation of FL PLK1.

**Fig. 7:**
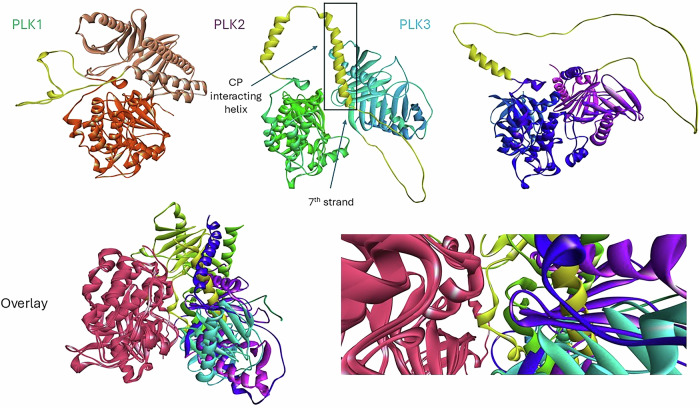
Comparison of AlphaFold models generated for PLKs 1–3. Individual PLK1s shown at the top of the figure are superimposed just using the KD residues to highlight the differences in the positions of the PBD in each case and this is further highlighted by the overlay figure. The compact autoinhibited PLK1 structure is not observed in PLK2 or PLK3. A significant finding from the PLK2 structure is the helical content of the IDL, its engagement with the CP and the 7^th^ strand of the β6α motif.

As described above, AF predictions provide significant insights into the structure of full-length PLK1 and confirm the autoinhibited structure first observed in the artificial Danio complex. As AF models of other PLKs exist with good confidence levels, the structures of FL PLK2 and 3 were examined by Wyatt and McInnes [90]. The striking observation was made that the compact autoinhibited conformation observed with PLK1 is not evident in the other family members (Fig. 7). As described above, while PLK1 has a plethora of hydrophobic interdomain contacts leading to the closed conformation, PLK2 and 3 have significantly fewer stabilizing interactions between the domains and those that exist are between residues containing polar side chains. This is apparent by comparing the residues that stabilize the PLK1 closed conformation in comparison with those for PLK2. While I408 is semi conserved as a F501, F409 is changed to Q502. L505, L546 and M547 of PLK1 are critical for stabilizing the autoinhibited state and their counterparts in PLK2 are the non-conservative replacements, R597, E639 and E640 respectively.

It is evident from these differences alone that a similar autoinhibited state for PLK2 is not likely. Furthermore, the PLK2 AF structure revealed several secondary structure elements that are not evident from the isolated KD or PBD crystal structures [90]. Relative to PLK1, it has a much larger IDL consisting of residues 350–454 and is predicted to contain two helical segments and a β-strand which forms the 7^th^ strand of a 6 strand anti-parallel β-sheet (Fig. 7). Furthermore, the loop connecting this strand with the first helix of the PBD possesses a salt bridge between E461 and K95 of the KD, bringing this loop in close proximity to the ATP binding site of the KD of PLK2. It does not however block access to the extent that the P403 containing loop interacts with the KD in PLK1. An interesting observation of this study is that the longer helical segment between residues 391 and 402 folds over the cryptic pocket of PLK2 and inserts two hydrophobic residues into this the tyrosine rich pocket (Fig. 7). This is intriguing given that the CP plays an important role in specific substrate recognition in the PLK1 context, it is highly conserved in PLK2 and therefore the IDL helix may play a regulatory role in preventing access to the cryptic pocket and phosphorecognition site. Furthermore, due to the conservation of the CP, it is a plausible scenario that this helix could facilitate heterodimerization between PLK1 and PLK2 previously observed [82].

Investigation of the AF structure of PLK3 suggests that it also does not adopt an autoinhibited conformation (Fig. 7). PLK3 has fewer interdomain contacts than PLK2 although there is some evidence of interactions between a loop of the PBD contacting the KD active site. K473 of this loop partially blocks access to the ATP binding site. In PLK3, the residues analogous to those stabilizing the closed conformation of PLK1 are not conserved, as with PLK2. I508 is L461 while F509 is V462. L505, L546 and M547 of PLK1 are V557, E599 and P600 respectively. Although the IDL of PLK3 is of similar length to that of PLK2 it has much less secondary structure according to the AF prediction (Fig. 7). One helical segment is observed covering residues 338-353 and the second helix contacting the PBD cryptic pocket in PLK2 and the 7^th^ β-strand are not predicted to occur with PLK3. The absence of the CP binding helix in PLK3 suggests the possibility that each of PLKs 1–3 has its own unique regulatory mechanism. This has not yet been investigated experimentally and therefore future studies could provide significant insights into these mechanisms.

**Fig. 8:**
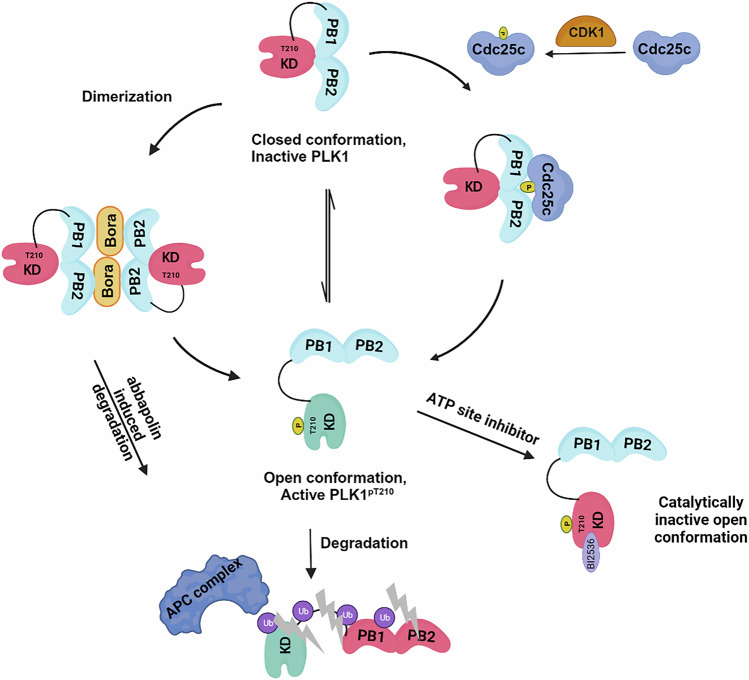
Model of the conformational regulation of PLK1. PLK1 is in an inactive state (red KD) when autoinhibited by a KD–PBD interaction or in a dimer promoted by PBD interactions. Binding of Bora relieves either form, but still retains PLK1 in an inactive state until recruitment of Aur-A which activates PLK1 through T210 phosphorylation (green KD). Phosphosubstrate binding has also been shown to lead to an increase in activity of PLK1. Monomeric PLK1 will be susceptible to degradation through exposure of the destruction box and Lys492 in the PBD (Fig. 1), Abbapolin PBD inhibitors have been shown to induce monomeric PLK1 and lead to their proteasomal degradation (left) [95]. ATP binding site inhibitors such as BI2536 induce a catalytically inactive open conformation of PLK1 [88, 89] (right).

Collectively, the structural analyses support the observations regarding the dimerization and of the open and closed conformational features of PLK1 (Fig. 8). Furthermore, analysis of the AF structures for PLKs1-3 indicates that they each have distinct mechanisms by which their activity is regulated. A major conclusion of Chapagai et al. [89] and Raab et al. [88] studies is that ATP-competitive compounds can induce a catalytically inhibited open conformation and therefore have the potential to increase affinity of PLK1 for its substrates. These observations have profound implications for drug discovery in that such compounds may promote non-catalytic functions of PLK1 and shed new light on the phenotypic responses observed following treatment with KD-binding inhibitors compared to PBD-binding inhibitors. Furthermore, this may have implications for selectively targeting PLK1 since binding would not be expected to lead to the same effects on PLKs 2 and 3. Indeed, these provide a rationale for simultaneous targeting of the ATP and PBD binding sites and hence for a combination treatment inhibiting both. Future studies aimed at elucidating the effects of intracellular conformational changes will help better define the basis for the interactions of PLK1 with itself, its substrates, and binding partners and further aid drug discovery efforts.

## Conclusions

In recent years, tremendous advances in understanding the regulatory mechanisms by which PLK1, a key mitotic kinase extensively investigated as a cancer drug target, have been achieved. The contributions and roles of the unique C-terminal PBD both to substrate recognition and to autoinhibition of the KD have been dramatically elucidated. Initial structural studies of the PBD revealed the basis for recognition of phosphorylated substrates primed by other kinases and how this contributed to subcellular localization of PLK1 to specific sites in mitosis. Identification of peptidomimetic PBD binding compounds containing alkylated histidine residues and subsequent structural analysis revealed the presence of a hydrophobic channel not present in the peptide structures. This tyrosine or cryptic pocket was later demonstrated to play a significant role in selective recognition of certain PLK1 substrates and through mutational analysis, its contributions to PLK1 functions identified. Furthermore, small molecule compounds that exclusively engage the CP were identified and provided insights into how selective inhibition of PLK1 substrates might be achieved. In addition to its recruitment roles, the PBD has been shown to have regulatory properties through its interactions with the KD and can adopt a kinase inactive autoinhibited state. Sequence and structural analysis suggests that PLKs 2 and 3 do not form the closed inactive state observed with PLK1 and this has significant implications for differential regulation and inhibition of their activity. Post-translational modifications including Thr210 phosphorylation and engagement with other proteins including Bora can also play regulatory roles in the timely control of PLK1 activity. PLK1 can undergo conformational transitions from the autoinhibited or closed structure to an open and active conformation in which the domain-domain inhibitory interactions are disfavored. Insights into this transition were obtained using ATP competitive compounds that preclude stabilizing interactions between the two domains and lead to a catalytically inhibited but open conformation. This conformation was shown to occur in cellular studies where a less stable PLK1 was observed after treatment of cells with BI2536 and other clinically evaluated ATP-competitive compounds. This inhibited open conformation has an increased affinity for PLK1 substrates therefore leading to the conclusion that while such compounds may block catalytic activity, they may simultaneously promote non-catalytic PLK1 activity that occurs through the PBD. This may at least partially explain the lack of efficacy of PLK1 inhibitors in clinical trials and justifies the combination of PBD targeted compounds with those blocking the ATP binding site. The recent results regarding FL PLK1 also merit revisiting drug development efforts regarding ATP-competitive, PBD-competitive and even allosteric binding molecules. Indeed, Park et al. recently reported an allosteric PBD targeted molecule called Allopole which is a prodrug of the active component Allopole-A. This compound binds to a previously undescribed pocket with high specificity by displacing a latch-like loop and as a result disrupts multiple interactions required for phospholigand binding to the canonical site. Blocking of PLK1 interactions with key substrate proteins through the PBD was shown to lead to its delocalization from centrosomes and kinetochores and ultimately promoted apoptosis [42].

Further insights into how PLK1 is regulated at the molecular level have been obtained through the identification of dimeric forms. One comprehensive analysis demonstrated that PLK1 is maintained in an inactive dimeric form throughout interphase of the cell cycle and then becomes monomeric just prior to being translocated into the nucleus at the end of the G2 phase of the cell cycle. This study revealed a key regulatory role for the monomer - dimer switch that functions in addition to the autoregulatory roles of the PBD. It is possible although not yet shown that the dimer provides an additional role in stabilizing the autoinhibited conformation of PLK1 and that transition to the monomer allows the conformational opening required for activity. The newly appreciated structural features of FL PLK1 may facilitate development of more potent inhibitors that better capture PLK1 in an inactive conformational state and prevent it adopting an open conformation that remains capable of binding substrates. Other modes of targeting such as PROTACs might also be developed to exploit conformational states. The class of molecules termed abbapolins described above can promote the loss of PLK1 in the absence of an E3-targeting ligand [95]. PBD targeted inhibitors represent a promising strategy to target PLK1 selectively and may even be useful combined with catalytic inhibitors, especially in light of the insights that these promote increased affinity to substrates.

Lastly, the identification of heterodimers of PLK1 with PLK2 opens up intriguing new insights into the regulation of the Polo-Like kinase family. The AF studies on PLK2 and 3 indicate that the different monomer conformations may have implications for the heterodimeric complexes also. While one PLK1 homodimer crystal structure has been solved, much remains to be discovered regarding the conformational regulation of PLK1 and continued biophysical, structural, and cellular studies should provide these insights in the near future. This information will shed light on the regulation of PLK1 activity and also will inform future drug discovery efforts targeting this important mitotic kinase. This also raises intriguing possibilities of better targeting the PLK family members besides PLK1. Beyond cancer, PLK2 and PLK3 have received attention in neurodegenerative diseases, because phosphorylation of synuclein at Ser129 is a hallmark of Parkinson disease and related synucleinopathies [96, 97]. Yet, there have been few published reports on the development and evaluation of selective small molecule inhibitors of PLK2 and PLK3. Newfound appreciation of the structural features of the PBD and FL PLKs 1–3 might translate into a better understanding how each members of the PLK family are regulated through conformational changes and/or dimerization (the catalytic site is highly conserved) and therefore offer new opportunities for their selective targeting.
